# Letter to the editor: Trends in tuberculosis notification rates by country of origin in the metropolitan area of Rome, 2010 to 2015

**DOI:** 10.2807/1560-7917.ES.2017.22.27.30570

**Published:** 2017-07-06

**Authors:** Monica Sañé Schepisi, Paola Scognamiglio, Maurizio D’Amato, Enrico Girardi, Vincenzo Puro

**Affiliations:** 1 Lazio Regional Service for the Surveillance of Infectious Disease (SeReSMI) Rome, Italy; 2 Clinical Epidemiology Unit, Lazzaro Spallanzani National Institute for Infectious Diseases, Rome, Italy

**Keywords:** tuberculosis incidence, foreign born, natives, Rome

**To the editor:** In their rapid communication, Hollo et al. [1] reported estimated trends in tuberculosis (TB) notification rates from 2010 to 2015 for 29 European Union and European Economic Area (EU/EEA) countries. They pointed out that the TB notification rate is decreasing at an annual rate of 5.3% and that this decrease was higher in native residents (7.0%) than in residents of foreign origin (3.7%).

The overall EU/EEA TB notification trend reported by Hollo et al. [1] reflects the fact that the specific migration trends of each country, including patterns of migration and bilateral net migration flows, differences in migration rate and differences in the proportion of individuals originating from a country with high incidence of TB, could not be taken into consideration [2,3]. In addition, integration patterns and social mixing may differ among EU/EEA countries and cities and define specific TB transmission dynamics. Finally, TB is known to concentrate in big cities as national incidence falls in middle to low incidence countries, most likely because of the higher concentration of risk groups there [4].

This widely heterogeneous pattern could not be shown by the analysis on national surveillance data by Hollo et al. [1], but it can be shown by other means. We hereby report that in Rome metropolitan area (MA), the TB notification rate in residents of foreign origin is declining faster than in native residents.

In 2015, the overall TB notification rate among Rome MA residents was 10.5 cases per 100,000 population, which was almost twice the national one for that year (6.2/100,000 population). In the period 2010 to 2015, a total of 1,030 TB cases were notified in native residents, with the number of cases per year ranging between 162 in 2015 and 186 in 2012. During the same period, a total of 1,901 TB cases were notified in residents of foreign origin, with the number of cases per year ranging between 295 in 2015 and 333 in 2011. From 2012 onwards, there has been a steady decrease in the total number of TB cases and in the overall notification rate, with an estimated average annual percentage change from 2010 to 2015 of −3.2% (95% confidence interval (CI): −6.0 to −0.3). However, this decrease was markedly different between native residents (−1.3%, 95% CI: −6.6 to 4.4) and residents of foreign origin (−12.7%, 95% CI: −16.1 to −9.1).

Two possible reasons may explain the differences between our results and those reported by Hollo et al. [1].

According to our data, the number of TB cases among residents of foreign origin decreased by 8.7% while the population of residents of foreign origin increased in size from 2010 to 2015 by 73.4%. This increase was not associated with changes in country of origin frequency distribution and it could be hypothesised that long-term settlement in Rome MA of new EU citizens could have progressively reduced the risk of developing TB. As per Hollo et al. [1], in EU/EEA countries from 2010 to 2015, a similar decrease in TB cases among residents of foreign origin (−7.3%) was observed, but the population increase of foreign born residents was much smaller (7.2%).

Furthermore, from 2012 onwards, an increasing number of residents of foreign origin in Rome MA and Italy migrated from countries with a TB notification rate lower than 100 per 100,000 population. This notification rate is lower than that of migrants to other European countries such as the United Kingdom, as reported previously in the literature [5]. It could be thus hypothesised that in comparison with Italy, EU/EEA countries with higher percentages of migrants from countries with a higher TB burden might have persistently higher TB notification rates among foreign born TB cases [6].

Our findings are consistent with those reported in Barcelona [7], another large city in the EU/EEA with large-scale immigration. Big city-specific data on TB incidence trends among native and foreign-born residents could help with understanding the interaction between migration and TB, and be decisive for TB control in the EU/EEA.
